# One-Year Radiologic Progression in Sporadic and Hereditary Cerebral Amyloid Angiopathy

**DOI:** 10.1212/WNL.0000000000213546

**Published:** 2025-04-08

**Authors:** Maaike C. van der Plas, Emma A. Koemans, Manon R. Schipper, Sabine Voigt, Ingeborg Rasing, Reinier G.J. van der Zwet, Kanishk Kaushik, Rosemarie van Dort, Sanne Schriemer, Thijs W. van Harten, Erik van Zwet, Ellis S. van Etten, Matthias J.P. van Osch, Gisela M. Terwindt, Marianne van Walderveen, Marieke J.H. Wermer

**Affiliations:** 1 Department of Neurology, Leiden University Medical Center, the Netherlands;; 2 Department of Radiology, Leiden University Medical Center, the Netherlands;; 3 Department of Biomedical Data Sciences, Leiden University Medical Center, the Netherlands; and; 4 Department of Neurology, University Medical Center Groningen, the Netherlands.

## Abstract

**Background and Objectives:**

Knowledge on the short-term progression of cerebral amyloid angiopathy (CAA) is important for clinical practice and the design of clinical treatment trials. We investigated the 1-year progression of CAA-related MRI markers in sporadic (sCAA) and Dutch-type hereditary (D-CAA).

**Methods:**

Participants were included from 2 prospective cohort studies. 3T-MRI was performed at baseline and after 1 year. We assessed macrobleeds, cerebral microbleeds (CMBs), cortical superficial siderosis (cSS), convexity subarachnoid hemorrhages (cSAHs), white matter hyperintensities (WMH), enlarged centrum semiovale perivascular spaces (CSO-EPVS), and visually stimulated blood oxygenation level–dependent (BOLD) fMRI parameters. Progression was defined as increase in number of macrobleeds or CMBs, new focus or extension of cSS, increase in CSO-EPVS category, or volume increase of >10% of WMH. Multivariable regression analyses were performed to determine factors associated with progression and the association between events related to parenchymal injury (cSAH, macrobleeds) and radiologic progression.

**Results:**

We included 98 participants (47% women): 55 with sCAA (mean age 70 years), 28 with symptomatic D-CAA (mean age 59 years), and 15 with presymptomatic D-CAA (mean age 45 years). Progression of >1 MRI markers was seen in all 83 (100%) participants with sCAA and symptomatic D-CAA and in 9 (60%) with presymptomatic D-CAA. The number of CMBs showed the largest progression in sCAA (98%; median increase 24) and symptomatic D-CAA (100%; median increase 58). WMH volume (>10% increase in 70%; mean increase 1.2 mL) was most progressive in presymptomatic D-CAA. A decrease in the upslope of the visually evoked BOLD response was observed for most patients. Symptomatic D-CAA status was associated with more overall progression (adjusted odds ratio [aOR] 9.7; 95% CI 1.7–54.2), CMB (adjusted relative risk [aRR] 2.47; 95% CI 1.5–4.1), and WMH volume progression (β 2.52; 95% CI 0.3–4.8). Baseline CMB count (aRR 1.002; 95% CI 1.001–1.002) was associated with CMB progression and cSS presence at baseline (aOR 8.16; 95% CI 2.6–25.4) with cSS progression. cSS progression was also associated with cSAH and macrobleeds (aOR 21,029; 95% CI 2.042–216.537).

**Discussion:**

CAA is a radiologically progressive disease even in the short-term. After 1 year, all symptomatic and most of the presymptomatic participants showed progression of at least 1 MRI-marker. CMBs and WMH volume (in symptomatic CAA) and WMH volume (in presymptomatic CAA) are the most promising markers to track short-term progression in future trials.

## Introduction

Cerebral amyloid angiopathy (CAA) is a common cerebral small vessel disease characterized by β-amyloid (Aβ) accumulation in the walls of leptomeningeal and cortical blood vessels.^1^ CAA is a major cause of lobar intracerebral hemorrhage (ICH) and a contributor to cognitive decline in the elderly.^2,3^ Several hemorrhagic MRI markers, such as cerebral microbleeds (CMBs) and cortical superficial siderosis (cSS), are well-known radiologic hallmarks of CAA.^4,5^ Over the recent years, there has also been increasing attention for nonhemorrhagic markers, such as enlarged perivascular spaces in the centrum semiovale (CSO-EPVS), white matter hyperintensities (WMHs), cortical microinfarcts, and vascular reactivity.^6-9^ This broad spectrum of (non)hemorrhagic markers has enhanced the diagnosis of CAA through the Boston criteria 2.0 and has increased our understanding of the pathophysiology of CAA. However, the value of these imaging markers for measuring short-term CAA progression for clinical and research purposes has yet to be determined.

Currently, the natural history of CAA on MRI is largely unknown. Most of what we know about the rate of disease progression in patients with CAA comes from retrospective or small prospective studies that focus on a singular marker of CAA (see eTable 1 for a summary of these studies).^10-16^ There are multiple promising potential therapeutic targets for the treatment of CAA, such as the production, aggregation, and clearance of Aβ, oxidative stress, and neuroinflammation.^17^ Although the prevention of lobar ICHs would be one of the main goals of any treatment, the incidental nature of these hemorrhages makes them unsuitable as a short-term outcome measure.^18^ For future trials, we require a CAA biomarker that is capable of accurately showing disease progression within a short time frame.

In this study, we investigated the 1-year evolution of CAA-related MRI markers in a unique cohort of participants with sporadic CAA (sCAA) and autosomal dominant Dutch-type hereditary CAA (D-CAA). D-CAA has an earlier onset and faster progression than sCAA but is otherwise pathologically and clinically similar.^19,20^ The advantage of including participants with D-CAA is that the presymptomatic disease phase can be studied in mutation carriers. As any future therapeutic strategy will most likely have to be implemented early in the disease course, this D-CAA subgroup will provide crucial information on disease progression in the earliest disease phases.

To increase our insight in the rate of progression of CAA and to identify potential short-term imaging outcome measures for future clinical trials, we aimed to determine (1) the 1-year radiologic progression of sCAA and D-CAA, (2) the imaging marker most capable of measuring short-term progression, and (3) factors associated with progression.

## Methods

### Study Participants

We included patients with CAA from 2 ongoing, natural history studies of the Leiden University Medical Center (LUMC): the AURORA study for D-CAA and the FOCAS study for sCAA. Both studies share the same study protocol in which patients are followed on a yearly basis with standardised research days (see the Supplements for a detailed overview of the study design). All patients were recruited through the (outpatient) clinic of the LUMC or communications via the D-CAA patient association. Inclusion criteria for D-CAA participants were age 18 years or older and presence of the causal amyloid precursor protein mutation. Presymptomatic mutation carriers and symptomatic (defined as a history of symptomatic ICH (sICH), transient focal neurologic episodes, and/or symptoms of cognitive decline interfering with daily life with a neuropsychological examination showing cognitive decline performed in the outpatient clinic as part of patient care) D-CAA patients were included. Patients with sCAA were included if they fulfilled the criteria for probable CAA according to the Boston criteria 2.0 and had no family history of D-CAA.^3^ Exclusion criteria included the inability to provide written informed consent. Participants were also excluded from parts of the study if they had specific contraindications to undergo 3T/7T MRI scans, lumbar punctures, or fMRI but could participate in parts of the protocol for which they had no contraindications. All participants underwent a research day at baseline and after 12 months. On both days, a 3T-MRI scan of the brain was performed and questionnaires regarding medical history and clinical symptoms were conducted. The following demographic and baseline characteristics were recorded: date of birth, sex, current medical conditions, daily intake alcohol/drugs/caffeine, smoking, medication, cardiovascular risk factors associated with other forms of small vessel disease (hypertension, hypercholesterolemia, diabetes type II, and atrial fibrillation), and neurologic history (previous ischemic or hemorrhagic stroke, seizures, and history of migraine). We included all patients with an MRI scan with at least susceptibility-weighted imaging (SWI) images at baseline and follow-up.

### Standard Protocol Approvals, Registrations, and Patient Consents

The local ethics review board of the LUMC approved the AURORA and FOCAS studies. All participants gave written informed consent before participation.

### Image Analysis

Participants underwent MRI's of the brain using the same whole body human 3T-MRI scanner (Philips Healthcare, Best, the Netherlands) at both time points (see Supplements for details on image acquisition).

In all participants, the presence, number and/or volume, and increase of CAA-related MRI markers were scored at baseline and follow-up by trained investigators (see eTable 2 for scored markers and used methods). We scored the following CAA-related MRI markers according to the STandards for ReportIng Vascular changes on nEuroimaging criteria: lobar macrobleeds, lobar CMBs, cSS, convexity subarachnoid hemorrhage (cSAH), WMH, and CSO-EPVS.^21^ For each participant, baseline and follow-up MRIs were compared by assessing the evolution of macrobleeds, CMBs, cSS, cSAH, WMH, CSO-EPVS, and time-to-peak (TTP), amplitude, and upslope as blood oxygenation level–dependent (BOLD) fMRI parameters.

Macrobleeds (defined as a hemorrhage ≥1 cm) and CMBs were assessed on SWI images using PACS Imaging Workstation (Sectra AB, Stockholm, Sweden) and subsequently marked using an in-house developed tool within the MeVisLab framework (version 3.2)^22^; MeVisLab is a flexible programming environment to design user interfaces that suit very specific image processing tasks, and the use of this tool allowed us to count large numbers of CMBs without risking double counts and compare scans from different timepoints slice by slice to analyze the occurrence of new bleeds (see Supplements for details). The use of MeVisLab did not influence the quality of the scan or the rating, resembling the rating on the original images, but with the added benefit of a tool to identify old and new bleeds more accurately. We chose not to score CMBs at follow-up that were also seen retrospectively (but unscored) on the baseline scan, as we consider these not to represent true CMB progression. sICH was defined as evidence of a recent ICH on imaging combined with focal neurologic symptoms corresponding to the location and approximate date of said ICH.

cSS was scored on SWI images according to location, focality,^23^ and cSS multifocality score.^24^ cSS contiguous or anatomically connected to a lobar ICH was not counted. If a hemorrhage developed near previously existing isolated cSS, we adhered to the baseline cSS score. cSS progression was scored for each participant according to the cSS progression score, as previously described.^11^ cSAH was scored on fluid-attenuated inversion recovery (FLAIR) imaging, based on previously described criteria.^23,25^

WMH were scored on FLAIR or T2 images, separately for periventricular and deep WMH using the Fazekas classification.^21,26^ In addition, WMHs were quantified on FLAIR images using an in-house developed semiautomatic segmentation pipeline in MeVisLab 3.61 (see supplementary material for details) and reported in milliliters. As the intrascan variability from various WMH volume quantification tools is estimated to be 10%–15%,^27^ we define volume increase of >10% in a participant at follow-up as progression (whereas in a sensitivity analysis, the threshold is set at 15%).

CSO-EPVS were scored on T2-weighted MRI using a validated visual rating scale.^28^ For each participant, the total CAA-related cerebral small vessel disease score (CAA-CSVD) was calculated at baseline and 1-year follow-up.^29^

Details on the processing of the BOLD data have been described elsewhere^30^ and can be found in the supplementary material. fMRI data processing was performed with FMRI Expert Analysis Tool^31^ v6.0, part of FMRIB's Software Library.^32^ We compared the BOLD parameters (TTP, amplitude and upslope) at baseline and follow-up. The BOLD upslope was calculated by dividing amplitude by TTP. When participants showed only a minimal response to the visual stimulus, no timing parameters could be calculated, and for these cases, only the amplitude parameter was included in the analysis.

The markers cSS, cSAH, qualitative WMH, and CSO-EPVS were scored by both observers for at least 20% of the MRIs at baseline and at follow-up, to prevent substantial interobserver variation. In case of macrobleeds, CMBs and quantitative WMH, at least 20% of the scores of the first observer were checked by a second observer. If a rater was uncertain, scans were discussed with a neuroradiologist with >15 years of experience in the field of neuroradiology. Scorers were not blinded to the condition of the participant (sCAA or D-CAA) nor to whether they were scoring a baseline or follow-up scan, as scans were scored side-by-side. Scorers were not aware of whether they were scoring a presymptomatic or a symptomatic D-CAA participant.

### Statistics

First, clinical characteristics of participants and frequencies of the CAA-related MRI markers at baseline and follow-up were reported using descriptive statistics. To assess within-participant longitudinal changes in MRI parameters, we reported proportions of the participants who had progression of one or more MRI markers and described progression per marker separately for sCAA, presymptomatic D-CAA, and symptomatic D-CAA, using the mean, median, and/or frequencies where appropriate. We also described the changes in CAA-CSVD and the overall progression (i.e., the amount of different markers showing progression per individual) between baseline and follow-up.

Second, we performed multivariable regression analyses to investigate possible predictors at baseline for progression of one or more structural markers (macrobleeds, CMBs, cSS, WMH >10%, and CSO-EPVS) and for individual markers (CMBs, cSS, and WMH volume) separately. We used an ordinal logistic regression model to calculate the adjusted odds ratio (aOR) for the number of markers showing progression (covariates: age, sex, type of CAA [sCAA, symptomatic D-CAA, presymptomatic D-CAA], sICH at baseline, use of anticoagulants/platelet inhibitors at baseline, and hypertension) and for the cSS severity progression score (additional covariate: cSS presence at baseline). We used a Poisson regression model to calculate the adjusted relative risk (aRR) for increase in number of CMBs (additional covariate: number of CMBs at baseline); due to overdispersion, we implemented Pearson χ^2^ as the method for estimating the scale parameter for the Poisson regression. A linear regression model was used for absolute increase in WMH volume (covariate in addition to the others: WMH volume at baseline instead of CMB count at baseline). As a post hoc analysis, we looked at the association of CMB and/or cSS progression with the occurrence of cSAH, sICH, or new macrobleeds during follow-up. These findings represent potentially significant decline in participants and are linked with the clinical course of CAA. We used binary logistic analysis to analyze whether the occurrence of such an event was associated with age and the amount of CMB increase or the cSS progression score (additional covariates: type of CAA, sex, sICH at baseline, hypertension, and use of anticoagulants/platelets). A *p* value <0.05 was considered statistically significant in all analyses.

### Data Availability

The data that support the findings of this study are available from the corresponding author on reasonable request.

## Results

**Figure 1:**
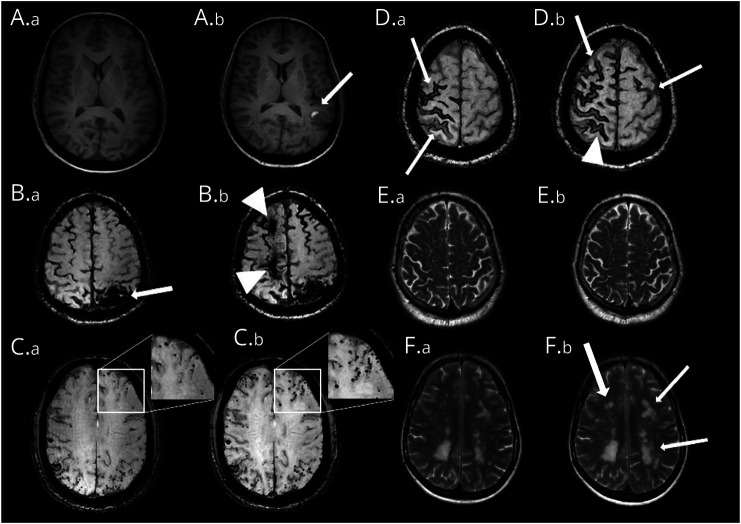
Examples of Markers at Baseline and Follow-Up Axial 3T-MRI scans showing examples of marker progression in different patients with CAA. (A) T1-weighted images showing the appearance of a recent symptomatic intracerebral hemorrhage in the left temporal lobe (A.b; arrow) of a patient with D-CAA at follow-up. (B) Susceptibility-weighted images showing a macrobleed in the left parietal lobe of a patient with sCAA at baseline (B.a; arrow) and the appearance of 2 more macrobleeds in the right frontal and frontoparietal lobe at follow-up (B.b; arrowheads). (C) Susceptibility-weighted images showing multiple CMBs in a patient with D-CAA at baseline (C.a) with an increase of CMBs at follow-up, particularly in the left frontal lobe (C.b; enhanced). (D) Susceptibility-weighted images showing cSS in the right frontal and parietal lobe at baseline in a patient with D-CAA (D.a; arrows) and at follow-up local extension of the cSS in the right hemisphere (D.b; arrowhead) as well as new foci of cSS in the right and left frontal lobes (D.b; arrows). (E) T2-weighted images showing category 4 (severe, >40 visible in 1 hemisphere) enlarged perivascular spaces in the centrum semiovale in a patient with D-CAA with no visible progression at follow-up. (F) T2-weighted images showing an increase of white matter hyperintensities in both hemispheres at follow-up (F.b, arrows) in a patient with D-CAA. CAA = cerebral amyloid angiopathy; CMB = cerebral microbleed; cSS = cortical superficial siderosis; D-CAA = Dutch-type hereditary CAA; sCAA = sporadic CAA.

At baseline, 67 participants with sCAA and 62 participants with D-CAA were screened. Twelve participants with sCAA and 19 with D-CAA did not undergo an MRI after 1 year for various reasons (follow-up not scheduled yet, morbidity or death, withdrew consent, lost to follow-up for unknown reasons, and claustrophobia) and were excluded; 1 participant was excluded because of excessive movement artefacts (eFigure 1). We included 98 participants with a median follow-up time of 12.0 months (interquartile range 11–12) in our final analysis: 55 with sCAA with a mean age of 70 years and 43 with D-CAA (28 symptomatic with a mean age of 59 and 15 presymptomatic with a mean age of 45 years, Table 1). eFigure 2 shows all sequences analyzed for the different markers. Figure 1 shows examples of marker progression.

**Table 1:** Baseline Characteristics

Baseline characteristics	sCAA (n = 55)	Symptomatic D-CAA (n = 28)	Presymptomatic D-CAA (n = 15)
Age at baseline, y, mean (range)	70 (58–84)	59 (47–74)	45 (35–55)
Male sex, n (%)	32 (54)	16 (57)	4 (27)
History of sICH at baseline, n (%)	25 (46)	23 (82)	0
Education >12 y, n (%)	32 (54)	14 (50)	9 (60)
Hypertension, n (%)	29 (49)	9 (32)	3 (20)
Hypercholesterolemia, n (%)	27 (46)	11 (39)	1 (7)
Diabetes type 2, n (%)	4 (7)	0	0
Smoking, n (%)			
Never	20 (34)	11 (39)	7 (47)
Smoking >1 y	35 (66)	17 (61)	8 (53)
Use of anticoagulants, n (%)	3 (6)	0	0
Use of platelet inhibitors, n (%)	9 (16)	0	0
MoCA at baseline, mean (SD)	24.8 (3)	26.5 (3)	27.4 (2)
Follow-up time, mo, median (range)	11.0 (10–19)	12.0 (11–17)	11.0 (11–14)

Abbreviations: CAA = cerebral amyloid angiopathy; D-CAA = Dutch-type hereditary CAA; MoCA = Montreal Cognitive Assessment; sCAA = sporadic CAA; sICH = symptomatic intracerebral hemorrhage.

### Progression of Hemorrhagic MRI Markers

#### sICH and Macrobleeds

Eight participants (1 [2%] with sCAA and 7 [25%] with symptomatic D-CAA) experienced in total 9 sICHs. Nineteen (19%) participants had an increase in the number of macrobleeds.

#### Cerebral Microbleeds

**Figure 2:**
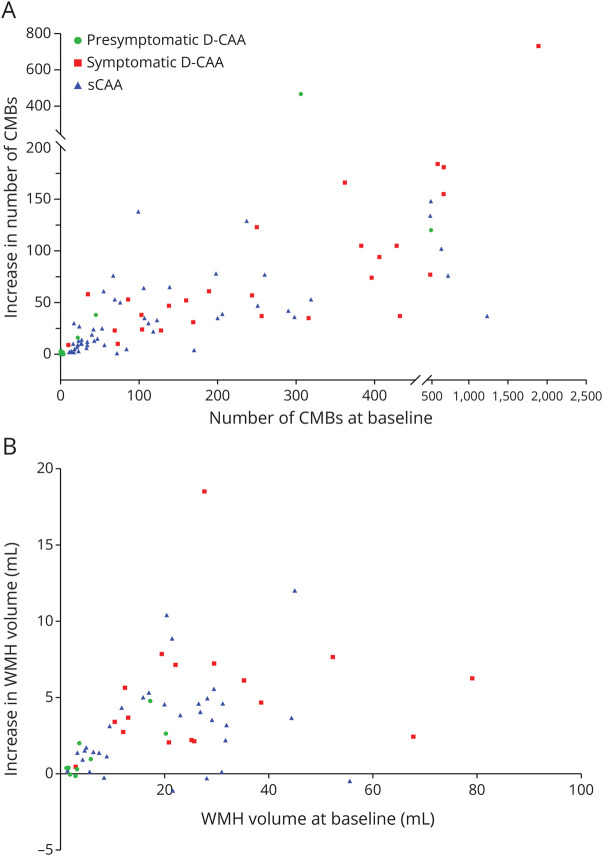
Increase of CMBs (A) and WMH Volume (B) Compared to Baseline CAA = cerebral amyloid angiopathy; CMB = cerebral microbleed; D-CAA = Dutch-type hereditary CAA; sCAA = sporadic CAA; WMH = white matter hyperintensity.

Eighty-seven (89%) participants had an increase in CMBs: 54 (98%) participants with sCAA and 5 (39%) participants with presymptomatic D-CAA. In both sCAA and symptomatic D-CAA, participants with a higher number of CMBs at baseline showed a larger absolute increase of CMBs (Figure 2A, eFigures 3 and 4A).

#### cSS and cSAH

Thirty (30%) participants showed cSS progression: 17 (31%) sCAA participants, 11 (39%) participants with symptomatic D-CAA, and 2 (13%) presymptomatic D-CAA participants. For the cSS progression scores, see eFigure 5.

None of the participants had cSAH at baseline. At follow-up, cSAH was seen in 2 (2%) participants with sCAA.

### Progression of Nonhemorrhagic MRI Markers

#### White Matter Hyperintensities

At baseline, 67 (68%) participants already reached the highest Fazekas category for periventricular WMH and 28 (29%) for deep WMH (eFigure 6). Between baseline and follow-up, there were no changes in Fazekas category in any participant.

Participants with a larger WMH volume at baseline showed a larger increase of WMH volume (Figure 2B, eFigure 4B). Forty-two (71%) participants had >10% increase of WMH volume (23 [72%] sCAA, 12 [71%] symptomatic D-CAA, and 7 [70%] presymptomatic D-CAA). There were no participants with a decrease in WMH volume of more than 10%.

#### Enlarged Perivascular Spaces in Centrum Semiovale

No participant changed CSO-EVPS category from baseline to follow-up (eFigure 7).

### Progression of Visually Stimulated BOLD fMRI Parameters

**Figure 3:**
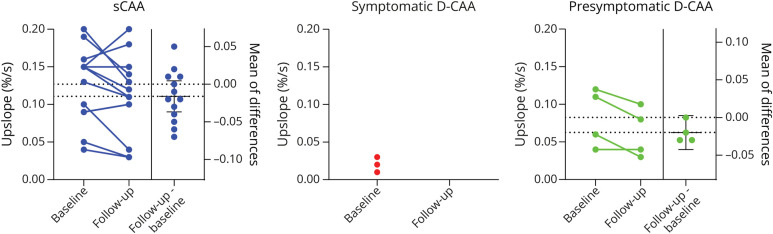
BOLD Parameter: Upslope BOLD = blood oxygenation level–dependent; CAA = cerebral amyloid angiopathy; D-CAA = Dutch-type hereditary CAA; sCAA = sporadic CAA

**Figure 4:**
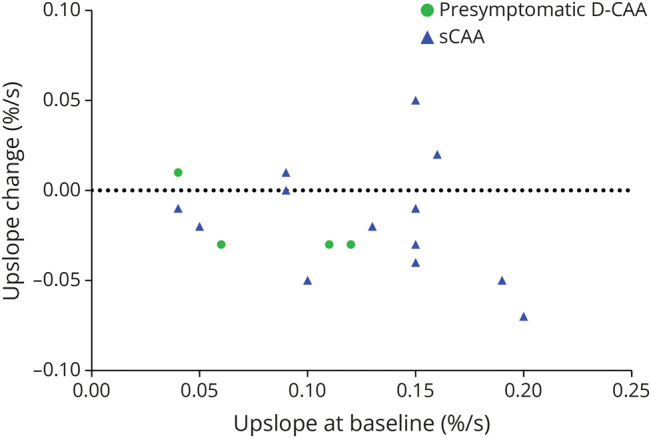
Decrease of BOLD Upslope Compared With Baseline BOLD = blood oxygenation level–dependent; CAA = cerebral amyloid angiopathy; D-CAA = Dutch-type hereditary CAA; sCAA = sporadic CAA.

The mean values of the TTP, amplitude, and upslope are presented in Table 2, whereas the trajectories of individual participants are shown in Figure 3 and eFigure 8. All 5 symptomatic D-CAA participants and 6 sCAA participants in whom we could assess the TTP of the BOLD response at baseline were considered no responders at follow-up (i.e., too small response to accurately estimate timing parameters). The change in TTP, amplitude, and upslope is presented in Figure 4 and eFigure 9.

**Table 2:** MRI Markers at Baseline and Follow-Up

	Baseline			Follow-up			Progression
	sCAA	Symptomatic D-CAA	Presymptomatic D-CAA	sCAA	Symptomatic D-CAA	Presymptomatic D-CAA	
Macrobleeds, median (IQR)	1.0 (0–2.0)	5.0 (2.0–10.8)	0	1.0 (0–2.0)	5.5 (3.0–11.8)	0	Participants with progression: 19 (19%) sCAA: 4 (7%; range 1–4) symp. D-CAA: 15 (54%, range 1–11) presymp. D-CAA: 0
Microbleeds, median (IQR)	56 (23–198)	253 (110–422)	0 (0–4)	78 (36–237)	326 (144–532)	0 (0–5)	Participants with progression: 87 (89%) sCAA: 54 (98%) symp D-CAA: 28 (100%) presymp D-CAA: 5 (39%)
cSS, n (%)	31/55 (56.3)	16/28 (57.1)	2/15 (13.3)	31/55 (56.3)	16/28 (57.1)	2/15 (13.3)	Participants with progression: 30 (30%) sCAA: 17 (31%) symp D-CAA: 11 (39%) presymp D-CAA: 2 (13%)
Disseminated cSS, n (%)	26/31 (83.9)	8/16 (50.0)	0/15	26/31 (83.9)	8/16 (50.0)	0/15	n/a
WMHs, Fazekas category, periventricular/deep, median (IQR)	3.0 (2–3)/2.0 (1–3)	3.0 (3–3)/2.0 (2–3)	1.0 (1–2)/1.0 (1–1)	3.0 (2–3)/2.0 (1–3)	3.0 (3–3)/2.0 (2–3)	1.0 (1–2)/1.0 (1–1)	No participants with progression in Fazekas category
WMHs volume, mean (SD)	20.4 (13.6)	29.1 (20.6)	5.9 (6.9)	23.6 (13.6)	34.4 (21.6)	7.1 (8.3)	Participants with >10% progression: 42 (71%) sCAA: 23 (72%) symp D-CAA: 12 (71%) presymp D-CAA: 7 (70%) Participants with >15% progression: 36 (61%) sCAA: 20 (63%) symp D-CAA: 10 (59%) presymp D-CAA: 6 (60%)
CSO-EPVS score, median (IQR)	4.0 (3.0–4.0)	4.0 (3.8–4.0)	4.0 (3.0–4.0)	4.0 (2–4)	4.0 (2–4)	4.0 (3.0–4.0)	No participants with progression in CSO-EPVS category
cSAH, n (%)	0/34	0/19	0/10	2/52 (3.8)	0/26	0/12	n/a
SVD CAA burden score, median (IQR)	5.0 (4.0–6.0)	5.0 (4.0–5.3)	1.0 (1.0–3.3)	5.0 (4.0–6.0)	5.0 (4.0–6.0)	1.0 (1.0–3.3)	Participants with progression: 5 (5%) sCAA: 2 (4%) symp D-CAA: 2 (8%) presymp D-CAA: 1 (7%)
TTP of BOLD response, s, mean (SD)	11.4 (4.9)	24.4 (4.5)	11.9 (2.4)	11.0 (4.8)	n/a	18.9 (6.6)	Mean difference: sCAA: 0.94 (SD 5.14) symp D-CAA: n/a presymp D-CAA: 7.00 (SD 6.39)
Amplitude of BOLD response, %, mean (SD)	0.87 (0.5)	0.45 (0.2)	0.85 (0.3)	0.82 (0.5)	0.58 (0.3)	0.96 (0.5)	Mean difference: sCAA: −0.046 (SD 0.37) symp D-CAA: 0.13 (SD 0.22) presymp D-CAA: 0.11 (SD 0.31)
Upslope of BOLD response in percentage per second, mean (SD)	0.1 (0.05)	0.02 (0.01)	0.08 (0.04)	0.1 (0.05)	n/a	0.06 (0.03)	Mean difference: sCAA: −0.017 (SD 0.034) symp D-CAA: n/a presymp D-CAA: −0.020 (SD 0.014)

Abbreviations: BOLD = blood oxygenation level–dependent; CAA = cerebral amyloid angiopathy; cSAH = convexity subarachnoid hemorrhage; CSO-EPVS = enlarged centrum semiovale perivascular spaces; cSS = cortical superficial siderosis; D-CAA = Dutch-type hereditary CAA; IQR = interquartile range; sCAA = sporadic CAA; sICH = symptomatic intracerebral hemorrhage; TTP = time-to-peak; WMH = white matter hyperintensity.

### Overall Progression of MRI Markers

**Figure 5:**
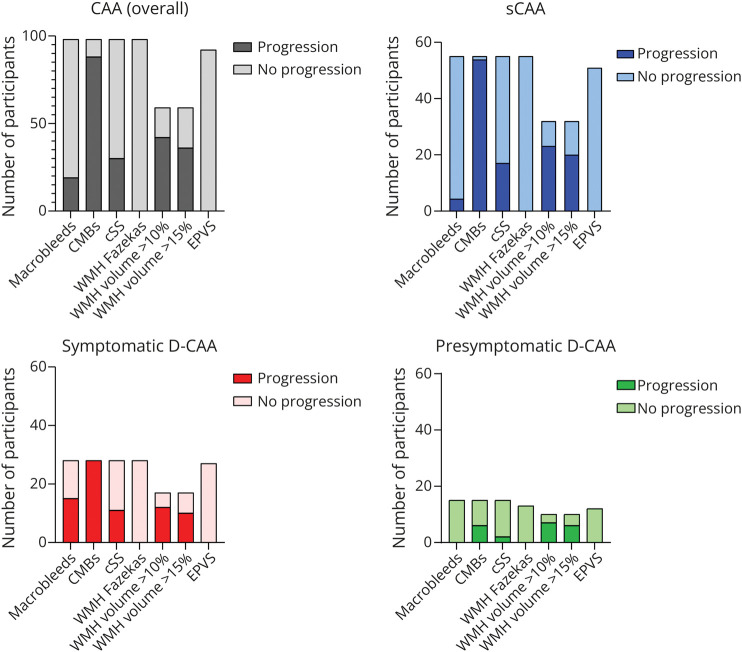
Number of Participants With Progression per Marker CAA = cerebral amyloid angiopathy; CMB = cerebral microbleed; D-CAA = Dutch-type hereditary CAA; EPVS = enlarged perivascular spaces; sCAA = sporadic CAA.

After 1-year follow-up, 92 (94%) of the 98 participants had progression of at least 1 CAA-related MRI marker out of macrobleeds, CMBs, WMH >10%, cSS, and CSO-EPVS (Figure 5 shows the proportion of participants with progression per marker; Table 2 presents the results per marker). All sCAA and all symptomatic D-CAA participants showed progression in ≥1 markers, as did 60% of presymptomatic D-CAA participants (eFigure 10).

The CAA-CSVD score increased between baseline and follow-up in 5 (5%) participants (eFigure 11).

### Factors Associated With Short-Term Progression on MRI

The results for our multivariable regression model analyses are summarized in eTable 3. Symptomatic D-CAA (aOR 9.7; 95% CI 1.74–54.2; *p* = 0.010) was the only covariate included in the model that was associated with the progression of more than 1 CAA-related MRI marker (out of macrobleeds, CMBs, cSS, WMH >10%, and CSO-EPVS).

Symptomatic D-CAA (aRR 2.47; 95% CI 1.50–4.09; *p* < 0.001) and the number of CMBs at baseline (aRR 1.002 per increase of 1 CMB; 95% CI 1.001–1.002; *p* < 0.001) were associated with CMB progression. Only the presence of cSS at baseline (aOR 8.16; 95% CI 2.62–25.4; *p* < 0.001) was associated with the cSS progression score. Symptomatic D-CAA (β 2.52; 95% CI 0.27–4.78; *p* = 0.028) was associated with WMH volume progression.

A cSS progression score of 4 (the highest category of cSS progression) was associated with the development of cSAH, new macrobleeds, or sICH (aOR 21,029; 95% CI 2.042–216.537; *p* = 0.010; eTable 3), whereas increase in CMBs was not.

For sCAA, symptomatic D-CAA and presymptomatic D-CAA analyzed separately (see eTable 4).

## Discussion

We found that progression of CAA can be identified with MRI even in the short term: all the participants with sCAA and symptomatic D-CAA and 60% of participants with presymptomatic D-CAA showed progression in macrobleeds, CMBs, cSS, or WMH volume during 1-year of follow-up. Having symptomatic D-CAA was associated with more overall progression, and the rate of progression of CMBs and WMH volume was higher in participants with D-CAA compared with sCAA. Our results suggest that CMB progression and WMH volume increase may be the most reliable markers to detect disease progression in advanced CAA, whereas WMH volume increase is the most valuable marker in the presymptomatic phase. This finding fits with our proposed pathophysiologic framework of CAA in which the disease evolves over several decades, from the initial vascular amyloid deposition (stage 1) and the alteration of cerebrovascular physiology (stage 2) to the appearance of nonhemorrhagic injury (stage 3), which precedes hemorrhagic injury (stage 4).^9^

CMB progression was seen in almost all sCAA participants and all symptomatic D-CAA participants. There was no apparent ceiling effect in our population: Participants with 500+ CMBs at baseline showed the largest absolute increase of CMBs. Only 1 participant with sCAA (with 1 CMB at baseline) had no increase in CMBs. Within our cohort, the number of CMBs at baseline is high compared with previous studies; we believe this is the result of our use of high quality MRI data combined with a reliable method of counting CMBs but also a consequence of being a specialized CAA centre, drawing the more severely affected patients to our clinic. Our data suggest that CMB progression is a promising radiologic marker in patients with more advanced CAA but will have less utility in the presymptomatic or early stage when patients have low CMB counts. The proportion of participants with CMB progression in our study was high compared with previous literature, and unlike other studies in CAA, we report the exact numbers of CMBs. A previous study reported CMB progression in 50% of 34 sICH survivors after 16 months (using 1.5T MRI) but was not aimed specifically at patients with probable sCAA.^33^ Higher percentages of CMB progression (67%–90%) at repeated imaging have been reported for sCAA as well, but the elapsed time between MRIs and the type of scanner was not stated, and the number of additional microbleeds was unknown.^34^ In a cohort of 79 patients with probable sCAA with a median follow-up time of 1.02 years, any CMB progression was seen in 70% and severe CMB progression (defined as >5 new CMBs) in 39% (using both 1.5T and 3.0T MRI), but the exact number of CMBs was not counted.^11^ In the same study, cSS progressed over 1 year in 29% of the 79 patients which is in line with our findings of cSS progression in 31% of sCAA participants. In presymptomatic D-CAA participants, cSS progression was limited, but not absent. Similar to our study, previous studies identified cSS presence at baseline as a strong prognostic factor for cSS progression.^11^

WMH volume increased more than 10% in 42 (71%) participants. A previous study reported a yearly median increase of WMH volume of 0.5 mL (18%) in patients with sCAA,^10^ while we found a median increase of 3.2 mL (19%). Although the proportion of increase was quite similar, the absolute increase in volume was higher in our study. In our study, participants also exhibited a larger WMH volume at baseline. This difference might be caused by the smaller sample size of the previous study with less affected patients including those with possible CAA. The volumes reported in our study seem to be comparable with the baseline values of several other large studies.^7,14^ Previous literature looking at absolute volume increase showed that WMH volume at baseline is strongly associated WMH progression at follow-up^10^; we only found this for the subgroups of sCAA and presymptomatic D-CAA (eTable 4).

Our data regarding BOLD TTP and amplitude showed substantial variation rather than consistent deterioration. A previous longitudinal study with 22 patients with sCAA has reported a decrease of BOLD amplitude at group level after 1 year, although the individual trajectories of the participants also varied considerably.^13^ Our data do show a decrease in the upslope at follow-up in most participants with BOLD data. The upslope has not been extensively investigated in patients with CAA but might be a promising parameter to explore further, as it combines both the TTP and amplitude. Our sample size of fMRI data was small compared with data on other markers, and there were several severely affected patients in whom no timing parameters could be assessed, as also demonstrated previously.^30^ Based on our findings, we can conclude that BOLD measures are less suitable for measuring progression in advanced D-CAA, as many patients will only show a minimal BOLD response at that point. Patients with less advanced disease and a relatively preserved BOLD response at baseline showed the most change at follow-up. Therefore, visually stimulated BOLD may be more suitable in assessing disease progression in less advanced disease stages. A larger sample size of presymptomatic D-CAA participants or sCAA participants in the early phase of the disease is needed to explore this.

The implication of our results is that short-term radiologic progression can be measured by structural markers in patients with CAA and that these markers—especially CMBs and WMH volume—can serve as outcome measures in clinical trials. The finding that most patients show increase in one or more MRI markers at short-term follow-up might also have clinical implications: The absence of any progression on high-quality 3T MRI imaging after yearly follow-up might indicate that the diagnosis of sCAA should be reconsidered. Future research will have to determine which of these radiologic markers most accurately reflect the disease process and the various symptoms of CAA. Thus far, the presence of CMBs and the CMB burden at baseline was found to be associated with an increased risk of future sICH in a study investigating cSS progression,^35^ but CMB progression itself has not been associated with future ICH risk^11^ nor is the number of CMBs linked to a worse trajectory of cognitive decline in patients with CAA.^36^ We could also not find an association between the increase in CMBs and events such as cSAH, new macrobleeds, or sICH during follow-up. The value of WMH increase in CAA needs to be more firmly established. WMH volume correlates with cognitive impairment in patients with lobar ICH and sCAA,^37^ but the connection to the hemorrhagic manifestations of CAA is less clear.^18^ Severe cSS progression has been linked to the increase of future ICH risk and is therefore a promising, albeit late, marker.^5,38^ Our data support the value of cSS progression as a predictor for events associated with parenchymal injury: The highest progression score was associated with the occurrence of cSAH, new macrobleeds, and sICH. cSS as a global marker for radiologic or clinical deterioration is complicated by the fact that it is not present in all patients and is a relatively late marker of CAA. This limits its usefulness as an outcome marker for future therapies. These therapies will most likely need to be implemented early in the disease course, when clinical events are limited and radiologic changes will be particularly relevant in measuring effectiveness.

Strengths of our study are our unique population that comprises probable sCAA, symptomatic D-CAA, and presymptomatic D-CAA, allowing us to study the sporadic version of the disease as well as the hereditary condition from the earliest phase to the later disease stages. Our data were collected and analyzed in a standardized manner, allowing a good comparison between the 2 time points. The use of 3T MRI and in-house tools to aid in the counting of hemorrhages and calculating WMH volume contributes to the reliability of our results. The systematic nature of our study, comprising various markers in different stages of the disease, contributes to our understanding of short-term CAA progression and illustrates the natural history of the disease. We hope that this increased insight in short-term progression will pave the way for measuring deviation from this course as the first therapeutic trials in patients with CAA will commence.

An important limitation of our study is that we did not relate our radiologic MRI marker progression to clinical decline. Clinical practice reveals that many patients do not exhibit signs or symptoms of decline, whereas they show progression of disease on imaging. Without treatment options, measuring radiologic progression does not benefit individual patients, although it may cause fear and anxiety. We are able to identify an increase in CMBs in almost all participants, but it is unclear what the clinical relevance of this finding is in participants with already high baseline numbers of CMBs. Other limitations of our study are the inherent bias that occurs in prospective follow-up studies, in which participants in the worst clinical condition are often the first to become lost to follow-up. This could have resulted in an underestimation of the severity of progression. Owing to the small group of presymptomatic participants with D-CAA (n = 15) and the exclusion of those younger than 35 years, our results for this group remain preliminary and limitedly generalizable. The presymptomatic group has low male representation (27%), which is believed to be due to chance rather than systematic bias. This is unlikely to affect the generalizability of the results, as a previous study found no substantial differences in MRI markers between men and women with D-CAA.^39^ Our selection of sCAA participants had a higher number of CMBs than in other CAA studies, which means that our results may not be reflective of patients with a lower disease burden. Furthermore, some of the outcomes we assessed (Fazekas score for WMH, qualitative scoring of CSO-EPVS, and CAA-CSVD score) showed substantial ceiling effects and therefore were unsuitable to measure longitudinal changes, underscoring the importance of moving toward quantitative scoring methods. CSO-EPVS may only be of value as categorical marker of progression in early stages of CAA, as even most of our presymptomatic participants were already in the highest categorical category of >40 EPVS. Current categorical assessment of EPVS could also lead to underestimation of the progression of CSO-EPVS. Even in this highest category, we do visually see large differences in CSO-EPVS count between participants (41–50 EPVS to far exceeding 100), which the score does not reflect. Without quantitative methods, we were not able to accurately measure progression of CSO-EPVS within 1 year. We hope this will change in the near future with the development of qualitative techniques.^40^ We used categorical scoring because it remains the most widely used approach in CAA studies, although our findings highlight its limitations for longitudinal research. Finally, longitudinal data on brain atrophy, cortical microinfarcts, and diffusion-tensor measurements were not yet available for our cohort.

In conclusion, we assessed short-term changes of structural radiologic markers in CAA and showed that progression in one or more MRI markers occurred in all of our sCAA and symptomatic D-CAA participants. The most promising structural markers for the use in clinical trials are CMBs for advanced CAA and WMH volume for both the early and advanced phase of the disease, while severe cSS progression may be associated with the occurrence of macrobleeds and cSAH in sCAA and symptomatic D-CAA participants.

## Glossary

Aβ: β-amyloid
aOR: adjusted odds ratio
aRR: adjusted relative risk
BOLD: blood oxygenation level–dependent
CAA: cerebral amyloid angiopathy
CAA-CSVD: CAA-related cerebral small vessel disease score
CMB: cerebral microbleed
cSAH: convexity subarachnoid hemorrhage
CSO-EPVS: enlarged centrum semi-ovale perivascular spaces
cSS: cortical superficial siderosis
D-CAA: Dutch-type hereditary CAA
FLAIR: fluid-attenuated inversion recovery
ICH: intracerebral hemorrhage
LUMC: Leiden University Medical Center
sCAA: sporadic CAA
sICH: symptomatic ICH
SWI: susceptibility-weighted imaging
TTP: time-to-peak
WMH: white matter hyperintensity
